# Dynamics of Influenza Seasonality at Sub-Regional Levels in India and Implications for Vaccination Timing

**DOI:** 10.1371/journal.pone.0124122

**Published:** 2015-05-04

**Authors:** Mandeep S. Chadha, Varsha A. Potdar, Siddhartha Saha, Parvaiz A. Koul, Shobha Broor, Lalit Dar, Mamta Chawla-Sarkar, Dipankar Biswas, Palani Gunasekaran, Asha Mary Abraham, Sunanda Shrikhande, Amita Jain, Balakrishnan Anukumar, Renu B. Lal, Akhilesh C. Mishra

**Affiliations:** 1 National Institute of Virology, Pune, India; 2 Centers for Disease Control and Prevention, Atlanta, USA; 3 Sheri-Kashmir Institute of Medical Sciences, Srinagar, India; 4 All India Institute of Medical Sciences, New Delhi, India; 5 National Institute of Cholera and Enteric Diseases, Kolkata, India; 6 Regional Medical Research Centre, Dibrugarh, India; 7 King Institute of Preventive Medicine & Research, Chennai, India; 8 Christian Medical College, Vellore, India; 9 Indira Gandhi Medical College, Nagpur, India; 10 King George Medical University (KGMU), Lucknow, India; 11 National Institute of Virology, Alappuzha, India

## Abstract

**Background:**

Influenza surveillance is an important tool to identify emerging/reemerging strains, and defining seasonality. We describe the distinct patterns of circulating strains of the virus in different areas in India from 2009 to 2013.

**Methods:**

Patients in ten cities presenting with influenza like illness in out-patient departments of dispensaries/hospitals and hospitalized patients with severe acute respiratory infections were enrolled. Nasopharangeal swabs were tested for influenza viruses by real-time RT-PCR, and subtyping; antigenic and genetic analysis were carried out using standard assays.

**Results:**

Of the 44,127 ILI/SARI cases, 6,193 (14.0%) were positive for influenza virus. Peaks of influenza were observed during July-September coinciding with monsoon in cities Delhi and Lucknow (north), Pune (west), Allaphuza (southwest), Nagpur (central), Kolkata (east) and Dibrugarh (northeast), whereas Chennai and Vellore (southeast) revealed peaks in October-November, coinciding with the monsoon months in these cities. In Srinagar (Northern most city at 34°N latitude) influenza circulation peaked in January-March in winter months. The patterns of circulating strains varied over the years: whereas A/H1N1pdm09 and type B co-circulated in 2009 and 2010, H3N2 was the predominant circulating strain in 2011, followed by circulation of A/H1N1pdm09 and influenza B in 2012 and return of A/H3N2 in 2013. Antigenic analysis revealed that most circulating viruses were close to vaccine selected viral strains.

**Conclusions:**

Our data shows that India, though physically located in northern hemisphere, has distinct seasonality that might be related to latitude and environmental factors. While cities with temperate seasonality will benefit from vaccination in September-October, cities with peaks in the monsoon season in July-September will benefit from vaccination in April-May. Continued surveillance is critical to understand regional differences in influenza seasonality at regional and sub-regional level, especially in countries with large latitude span.

## Background

Effective influenza surveillance systems are essential to understand the epidemiology and seasonality of influenza and for optimizing influenza control strategies. Influenza occurs in distinct outbreaks of varying extent every year.[1,2] This epidemiologic pattern depends upon multiple factors, including transmissibility of the virus and the susceptibility of the population.[3,4] In temperate regions of the Northern and Southern Hemispheres (NH and SH), influenza peaks during respective winter months, whereas the pattern of influenza varies in tropical and subtropical regions.[5–8] The seasonal fluctuations in environmental and social factors have been associated with the complex seasonality and transmission of influenza around the world. [9,10] While the underlying cause of the variable nature of seasonality for influenza in tropical countries remains elusive, indoor crowding, lower temperatures, and decreased humidity at a given latitude may influence both transmission and host susceptibility. [4,9–12] These studies suggest a paradigm shift for influenza seasonality for countries in northern hemisphere.[9]

Given the diverse topography and climatic conditions in various parts of India, a systematic laboratory-based surveillance of influenza viruses has been carried out in geographically distinct regions in India. Initial observations with limited sites revealed major peaks of influenza coinciding with the rainy season in the sub- tropical region of India in Pune, Delhi, Kolkata and Chennai[13,14] though some level of circulation was observed throughout the year. In the current study, we summarize data on influenza surveillance from distinct parts of India which identified varying seasonality, with unpredictability of emergence of circulating types and subtypes. These data highlight the need to revisit latitude dependence for influenza vaccination timing for the Asia region.

## Materials and Methods

### Study sites

Influenza Network in India is comprised of ten sentinel sites strategically located to cover major areas of India. The participating centers and the states from north to south were Sheri-Kashmir Institute of Medical Sciences (SKIMS), Srinagar (Jammu and Kashmir State, northernmost India, 34.0°N); All India Institute of Medical Sciences (AIIMS), New Delhi (Delhi, North India, 28.6°N); Regional Medical Research Center (RMRC), Dibrugarh (Assam, North-east, 27.5°N), King George Medical University (KGMU), Lucknow (Uttar Pradesh, North-central, 26.8°N); National Institute for Cholera and Enteric Diseases (NICED), Kolkata (West Bengal, Eastern India, 22.6°N); Indira Gandhi Medical College (IGMC), Nagpur (Maharashtra, Central India; 21.2°N); National Institute of Virology(NIV), Pune (Maharashtra, Western India, 18.5°N); Christian Medical College and Hospitals (CMCH), King Institute of Preventive Medicine (KIPM), Chennai (Tamil Nadu, South India, 13.1°N); Vellore (Tamil Nadu, South, 12.9°N) and National Institute of Virology, Alappuzha (Kerala, southern-most India, 9.5°N). Surveillance was carried out mostly among patients presenting to outpatient departments (OPD) with influenza like illness (ILI) and few sites for severe acute respiratory illness (SARI) surveillance in hospitalized patients. NIV, Pune was the referral center for the entire study. [13] Each center was required to randomly collect 5–10 specimens per week throughout the study period.

### Case Definition of ILI and SARI

A person presenting with sudden onset of fever >38° C or history of sudden onset of fever in the recent past (less than three days), and cough or sore throat or rhinorrhea.[15] SARI was defined as an ILI case with breathlessness or difficulty in breathing/tachypnea or clinically suspected pneumonia (in children) with increased respiratory rates as per Integrated Management of Childhood Illness.

### Laboratory Diagnosis

Combined throat and nasal swabs were collected in viral transport media and transported to the virology laboratory on ice within 4 hours of collection.[13,15] All samples were tested by real- time RT-PCR for the detection of influenza viruses using the Centers for Disease Control and Prevention protocol.[15] All seasonal influenza A positive samples were further sub-typed for A/H1 and A/H3.[15] A confirmed case was defined as a patient meeting the ILI/SARI case definition and positive for influenza by RT-PCR.

### Sequencing and Phylogenetic Analysis

Haemagglutinin 1 (HA-1)and NA genes were sequenced and amplicons were purified using PCR purification kits (Qiagen). The sequencing was done on ABI 3730 DNA analyzer and pair wise sequence alignment and Neighbour-joining (N-J) tree was generated using pair-wise gap deletion, maximum composite likelihood using Tamura-Nei nucleotide model in MEGA version 4.[16] All sequences were compared with published cognate sequences of corresponding genes from global data base, including those from India.[14,17]

### Statistical Analysis

Data on laboratory-confirmed influenza for each centre were entered in MS Excel (Microsoft, Redmond, United States of America) and PASW Statistics 18 (SPSS Inc., Chicago, USA). Monthly influenza activity was calculated by adding the weekly number of specimens that tested positive during a given month. The monthly data were then plotted as the percentage of all positive specimens during the calendar year that corresponded to that month. Means and standard errors were calculated from the cumulative data for each centre over the period evaluated. Monthly average data on maximum and minimum temperatures, relative humidity and rainfall were collected from the Governmental Meteorological Departments by all centers. Monthly influenza activity was compared with meteorological variables for linear correlation by bivariate analysis and plotted against monthly meteorological data to study seasonal patterns.

## Results

### Circulating Influenza strains in India

Due to the initiation of influenza surveillance at different years in the surveillance network, data was available for Delhi, Dibrugarh, Kolkata, Pune, Chennai, and Vellore from 2009–2013 and from Srinagar, Lucknow, Nagpur, and Alappuzha from 2011–2013. Of the total of 44127 specimens tested from the ten cities in India, 8371 (14.0%) were positive for influenza (Table 1), 69.0% of which were influenza type A and 31.0% influenza B. Of type A influenza, A/H1pdm09 accounted for 50.3% and A/H3 for 48.0%, with some circulation of seasonal H1 (1.7%) prior to the pandemic in 2009. City-specific data are summarized below.

**Table 1:** Influenza positivity from 10 sentinel surveillance sites in India.

City, State		2009	2010	2011	2012	2013	Total
**Srinagar, Jammu & Kashmir**	Sample Tested	NA	NA	771	1414	1735	3920
	Influenza Positives			162 (21.2%)	238 (16.8%)	305 (17.6%)	705 (18.0%)
**Delhi,**	Sample Tested	1053	663	1006	1511	1338	5571
	Influenza Positives	311 (29.5%)	103 (15.5%)	76 (7.6%)	145 (9.6%)	174 (13.0%)	809 (14.5%)
**Dibrugarh, Assam**	Sample Tested	642	731	599	695	242	2909
	Influenza Positives	111 (17.3%)	89 (12.2%)	74 (12.4%)	156 (22.4%)	34 (14.0%)	464 (16.0%)
**Lucknow, Uttar Pradesh**	Sample Tested	NA	NA	951	1776	1257	3984
	Influenza Positives			94 (9.9%)	340 (19.1%)	118 (9.4%)	552 (13.9%)
**Kolkata, West Bengal**	Sample Tested	455	896	1038	2211	962	5562
	Influenza Positives	72 (15.8%)	181 (20.2%)	242 (23.3%)	206 (9.3%)	63 (6.5%)	764 (13.7%)
**Nagpur, Maharashtra**	Sample Tested	NA	NA	590	917	1172	2679
	Influenza Positives			29 (4.9%)*	99 (10.8%)	165 (14.1%)	293 (10.9%)
**Pune, Maharashtra**	Sample Tested	1746	1298	853	1029	962	5888
	Influenza Positives	391 (22.4%)	279 (21.5%)	142 (16.6%)	95 (9.2%)	85 (8.8%)	992 (16.8%)
**Chennai, Tamil Nadu**	Sample Tested	18821**	7811**	1523	1291	751	5959
	Influenza Positives	1718 (9.1%)	667 (8.5%)	208 (13.7%)	104 (8.1%)	55 (7.3%)	560 (9.4%)
**Vellore, Tamil Nadu**	Sample Tested	482	399	543	1021	587	3032
	Influenza Positives	92 (19.1%)	72 (18%)	80 (14.7%)	163 (16%)	127 (21.6%)	534 (17.6%)
**Alappuzha, Kerala**	Sample Tested			824	2457	1342	4623
	Influenza Positives	NA	NA	154 (18.4%)	215 (8.8%)	137 (10.2%)	506 (10.9%)
**All Centers Total**	Sample Tested	23199	11798	8698	14322	10348	44127
	Influenza Positives	2695(11.6%)	1391 (11.8%)	1261 (14.5%)	1761 (12.3%)	1263 (12.2%)	8371 (14%)

* Influenza was detected by isolation in MDCK cells.

** A total of 24231 specimens were tested only for influenza A during 2009–2010 due to pandemic surge in testing capacity.

#### Srinagar

**Fig 1:**
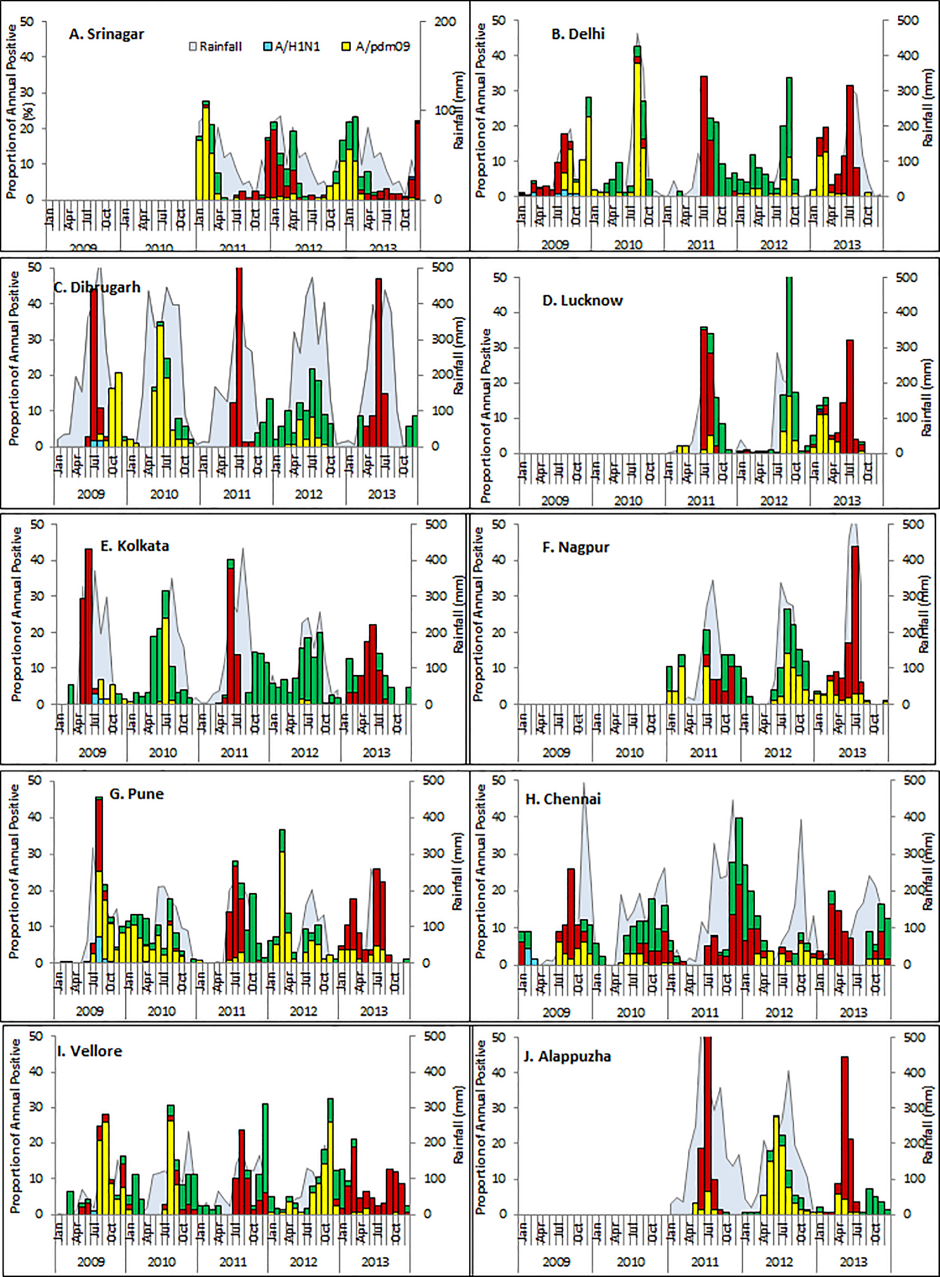
Monthly trends and seasonality of circulating influenza viruses in India, 2009–2013. Data is shown for Srinagar (A), Delhi (B), Dibrugarh (C), Lucknow (D),Kolkata (E), Nagpur (F), Pune (G), Chennai (H), Vellore (I), and Alappuzha (J). The left axis shows the proportion positive for influenza A/H1N1 (blue), A/H1N1pdm09 (yellow), A/H3N2 (red) and influenza B (green). The total number of influenza positives in a year were considered as 100%, and the percent positivity for each month was calculated for each year. Rainfall is shown in the background.

**Fig 2:**
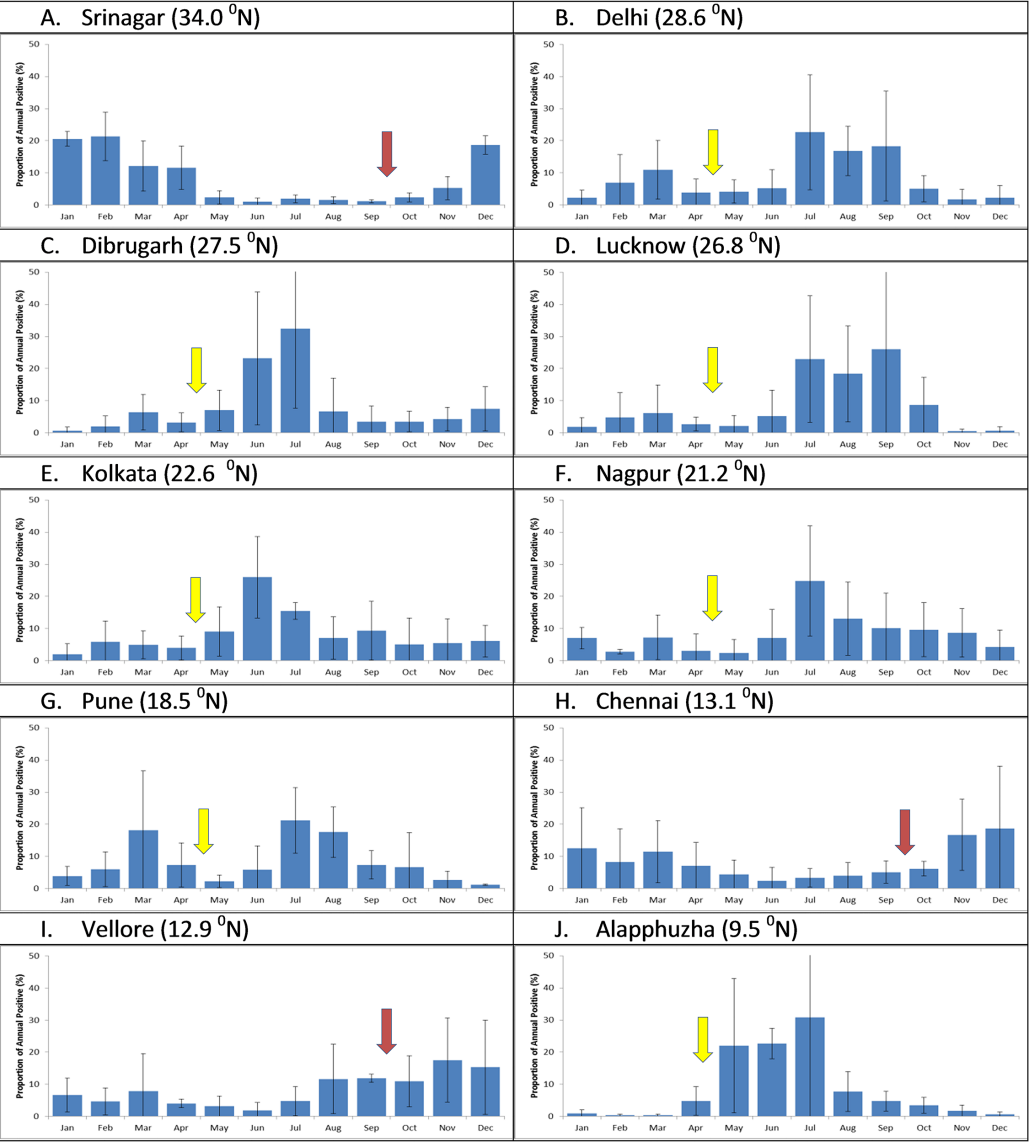
The proportion of influenza virus positivity by year (2009–2013) in India. Cumulative data on monthly distribution of influenza viruses by city was calculated (data shown is Mean± 1 SE). For this analysis, data from 2009–2010 was excluded due to the pandemic which did not follow the usual seasonality pattern. Arrow indicates proposed vaccination timing during September-October (red arrow) or April-May (yellow arrow). The latitude for the capital city of each country is shown on the top of each panel.

**Fig 3:**
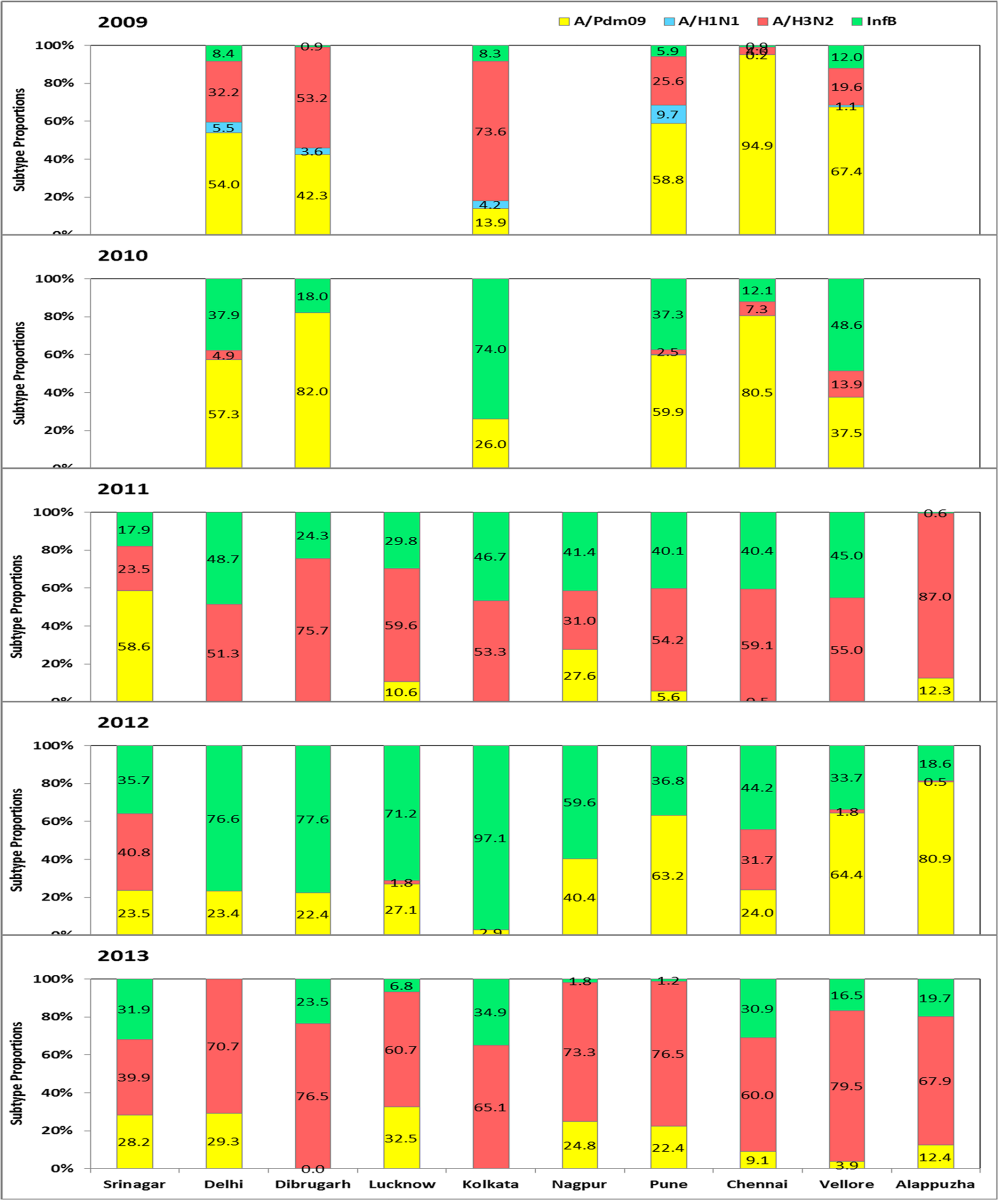
Influenza virus type and subtype distribution by year and city. The overall % positivity of types and subtypes is shown for each city. The left axis shows the percent monthly distribution of seasonal influenza A/H1 (blue); A/H3 (red), influenza B (green) and pandemic A/H1N1pdm09 (yellow) from 2009 to 2013.

705 of 3920 specimens (18.0%) were positive for influenza viruses (Table 1). Analysis of monthly data over a three-year period showed influenza circulation primarily from December-April for most years (Fig 1A), with discrete peaks in January-March (Fig 2A). The predominant subtype was influenza A/H1N1pdm09 in 2011; whereas A/H3 and influenza B co-circulated during 2011–2013 (Fig 3).

#### Delhi

809 of 5571 specimens (14.5%) were positive for influenza viruses (Table 1). Analysis of monthly data over a five period showed influenza circulation primarily from June-October for most years (Fig 1B), with discrete peaks in July-September during four of the study years (Fig 2B). The predominant subtype was influenza A/H1N1pdm09 in 2009, and 2010; A/H3 in 2011 and 2013; and influenza B in 2012 (Fig 3).

#### Dibrugarh

464 of 2909 specimens (16%) were positive for influenza viruses (Table 1). Analysis of monthly data over a five period showed influenza circulation primarily from May-August for most years (Fig 1C), with discrete peaks in June-July over four of the study years (Fig 2C). The predominant subtype was influenza A/H1N1pdm09 in 2009, and 2010; A/H3 in 2011 and 2013; and influenza B in 2012 (Fig 3).

#### Lucknow

552 of 3984 specimens (13.9%) were positive for influenza viruses (Table 1). Analysis of monthly data over a three-year period showed influenza circulation primarily from July-October for most years (Fig 1E), with discrete peaks in August-September over three years study period (Fig 2E). The predominant subtype was influenza A/H3 in 2011 and 2013; and influenza B in 2012 (Fig 3).

#### Kolkata

764 of 5562 specimens (13.7%) were positive for influenza viruses (Table 1). Analysis of monthly data over a five period showed influenza circulation primarily from May-August for most years (Fig 1D), with discrete peaks in June-July over four of the study year (Fig 2D). Surprisingly, influenza A/H1N1pdm09 was not the major circulating subtype; the predominant subtype was influenza A/H3 in 2009, 2011 and 2013; and influenza B in 2010 and 2012 (Fig 3).

#### Nagpur

293 of 2679 specimens (10.9%) were positive for influenza viruses (Table 1). Analysis of monthly data over a three-year period showed influenza circulation primarily from July-November for most years (Fig 1F), with discrete peaks in July-September (Fig 2F). The predominant subtype was influenza A/H3 in 2013; and influenza B in 2012 and 2012 (Fig 3).

#### Pune

992 of 5888 specimens (16.8%) were positive for influenza viruses (Table 1). Analysis of monthly data over a five period showed influenza circulation primarily from June-October for most years (Fig 1G), with discrete peaks in July-September over four years (2009–2010 data excluded) study period (Fig 2G). The predominant subtype was influenza A/H1N1pdm09 in 2009, 2010 and 2012; A/H3 in 2011 and 2013 (Fig 3).

#### Chennai

560 of 5959 specimens (9.4%) were positive for influenza viruses (Table 1). Analysis of monthly data showed influenza circulation primarily from September to November for most years (Fig 1H), with discrete peaks in September to December over three years (2009–2010 data excluded) (Fig 2H). The predominant subtype was influenza A/H1N1pdm09 in 2009 and 2010; A/H3 in 2011 and 2013; and influenza B in 2012 (Fig 3).

#### Vellore

376 of 3133 specimens (14.3%) were positive for influenza viruses (Table 1). Analysis of monthly data showed influenza circulation primarily from September-November for most years (Fig 1I), with discrete peaks in August to December (2009–2010 data excluded) study period (Fig 2I). The predominant subtype was influenza A/H1N1pdm09 in 2009, 2010 and 2012; A/H3 in 2011 and 2013; and influenza B in 201 2 (Fig 3).

#### Alappuzha, Kerala

506 of 4623 specimens (10.9%) were positive for influenza viruses (Table 1). Analysis of monthly data showed influenza circulation primarily from May-August for most years (Fig 1J), with discrete peaks in June-July (Fig 2J). The predominant subtype was influenza A/H1N1pdm09 in 2012; and A/H3 in 2011 and 2013 (Fig 3).

### Influenza seasonality and vaccination timing

**Fig 4:**
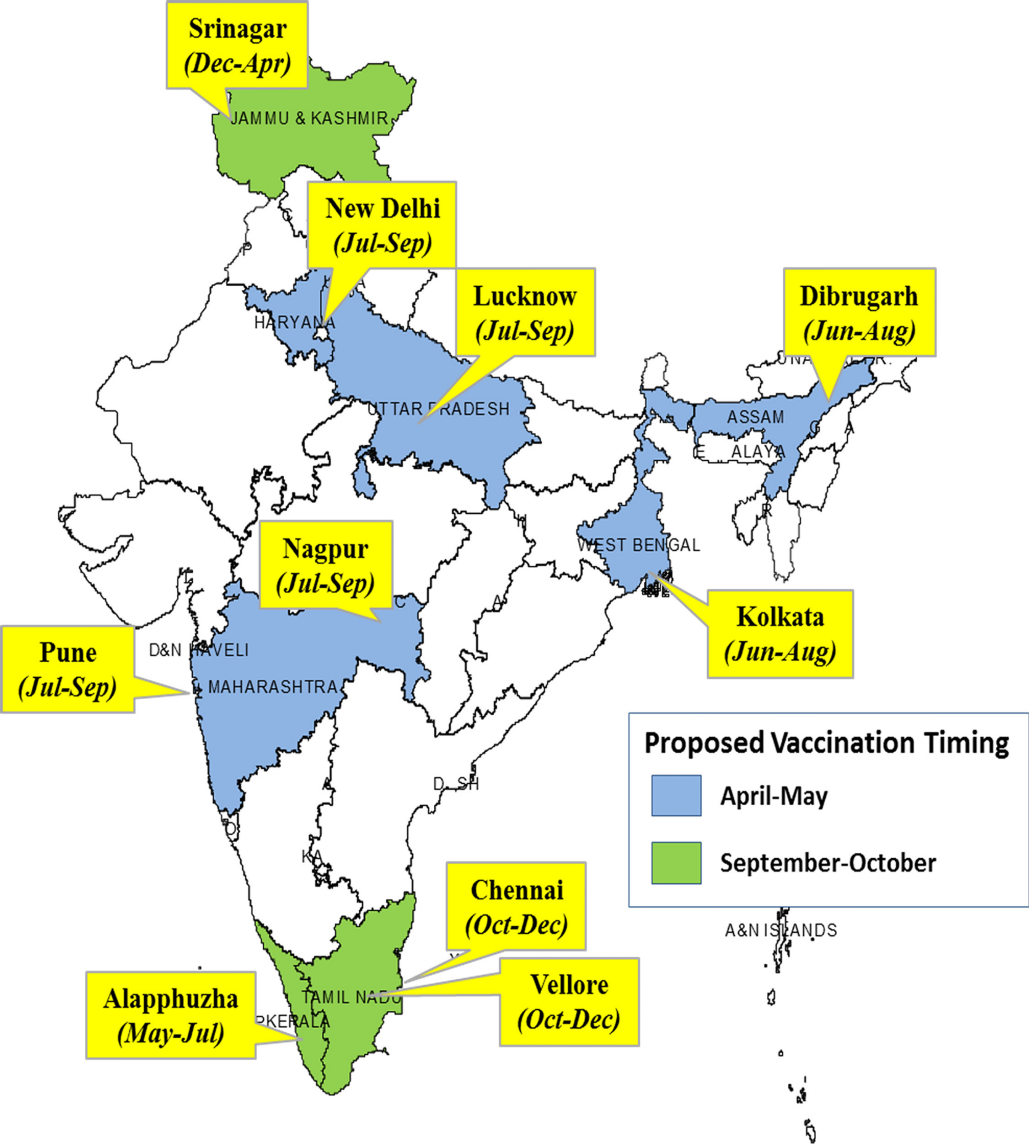
Peak influenza circulation based on seasons in India. Flags show names of the city, and peaks months of influenza circulation. India map is color coded for states with proposed vaccination timing with blue color for April-May and green color used for September-October.

Three major patterns of influenza circulation can be seen (Fig 2): peak influenza circulation during winter in Srinagar (January-April), peak influenza activity from June-October in Delhi, Dibrugarh, Lucknow, Kolkata, Nagpur, Pune, and Alappuzha, with minor peaks during winter time and a late monsoon related peaks in Chennai and Vellore from September to December. Cumulative analysis over the years revealed that >86% of influenza positive cases occurred during December-May for Srinagar. Further, >60% of influenza cases were observed in June-November for most other cities with the exception of Chennai and Vellore (Table 2);they had their highest influenza circulation during November-December, associated with the late monsoon. Influenza seasonality reveals a very complex pattern at the sub-regional level as shown in Fig 4.

**Table 2:** Relationship between geographic location, influenza seasonality, and proposed vaccination timing.

City, State	Latitude	Peak Seasonality	Proportion of Influenza Positive (%)		Proposed Vaccination
			June-November	December-May	
Srinagar, Jammu and Kashmir	34°0	Dec-Feb	13.3	86.7	Sep-Oct
Delhi	28°6	Jul-Sep	69.7	30.3	Apr-May
Dibrugarh, Assam	27°5	Jun-Jul	73.4	26.6	Apr-May
Lucknow, Uttar Pradesh	26°8	Jul-Sep	81.6	18.4	Apr-May
Kolkata, West Bengal	22°6	Jun-Jul	68.2	31.8	Apr-May
Nagpur, Maharashtra	21°2	Jul-Aug	73.3	26.7	Apr-May
Pune, Maharashtra	18°5	Jul-Aug	61.2	38.8	Apr-May
Alappuzha, Kerala	9°5	May-Jul	70.9	29.1	Apr-May
Chennai, Tamil Nadu	13°1	Nov-Dec	*37*.*7*	*62*.*3*	Sep-Oct
Vellore, Tamil Nadu	12°9	Nov-Dec	*58*.*4*	*41*.*6*	Sep-Oct

### Circulating influenza types and subtypes

In addition to seasonal variation, circulating types and subtypes also showed variation across cities in a given year (Fig 3). Influenza A/H3 predominated prior to emergence of pandemicH1N1pdm09 in May-June in most cities in India. Influenza A/H1N1pdm09 and influenza B viruses were the predominant strains in 2010, and A/H3 and influenza B co-circulated in 2011; however, their proportion varied from city to city. In 2012, influenza B was the dominant strain with some circulation of A/H1N1 pdm09. In 2013, H3 emerged as the dominant strain with co-circulation of A/H1N1pdm09. Of note, the predominant circulating strains in the Delhi area during the monsoon season (June-August) were usually the dominant strain in Srinagar the following winter. The antigenic and genetic analysis of circulating strains did not reveal any significant change between cities (S1 and S2 Tables, S1 Fig)

### Influenza seasonality and Latitude differences

Geographically, India falls in the tropical region between the equator and Tropic of Cancer (23.4°N) and the subtropical region with latitude less than 40°N. We examined the relationship between influenza positivity and the latitudes of the capital city of each state. Srinagar at the latitude of 34°N had an influenza peak in the winter, whereas most of the cities a latitude <30°N had influenza peaks during summer monsoon months (July- September), with Chennai and Vellore located at the south-west location have peaks in November-December (Table 2). Rainfall correlated with influenza peaks in all cities except Srinagar (P<0.05; data not shown)

## Discussion

Influenza epidemics in India show seasonal variations at sub-regional levels. We identified three discrete patterns: cities with temperate seasonality with peaks in December-March (Srinagar); cities with an influenza peak in July-October (Delhi, Dibrugarh, Lucknow, Kolkata, Pune, and Alappuzha) with additional winter peaks (Delhi, Nagpur, Pune); and those with late monsoon weather (Chennai and Vellore) with peaks during November-December. The seasonality of influenza in India appears to depend on geographic location (latitude) in addition to rainfall and possibly other environmental factors such as humidity. These findings highlight the need for expanded surveillance to understand complexity of influenza seasonality in a diverse country with varied climatic factors and topography.

In the current study, Delhi, Dibrugarh, Lucknow, Kolkata, Nagpur, Pune, and Alappuzha, have influenza peaks that coincide with rainy season during July-September, Chennai and Vellore have peaks coinciding with late monsoon, whereas Srinagar region influenza peaks coincides with cold temperature and low dew points. Several direct and/or indirect environmental factors are thought to drive the seasonality of influenza; including indoor crowding during cold and wet seasons, increased virus survival in cold and dry conditions, decreased immunity of the host, perhaps mediated by a decrease in Vitamin D synthesis from lack of sunlight during winter months. [1,2,18,19] While cold temperatures, low indoor humidity and minimal solar radiation have been associated with a higher activity of influenza in temperate regions, a link between increased influenza activity and high humidity in the rainy season in several tropical populations has also been reported.[19] The peak of influenza during winter in Srinagar is similar to influenza circulation observed for most countries in Europe and United States in northern hemisphere. [3] The data presented here corroborate recent findings, where pattern of influenza peaks appear different in tropical and subtropical areas, with annual peaks in some countries and year round circulation in others.[5–9] In addition to climatic drivers of influenza seasonality, geographic factors play a role in influenza seasonality. In the current study, we observed winter peaks of influenza circulation in Srinagar (30°N), whereas those below approximately 30°N latitude have seasonality coinciding with monsoon. A recent study from China revealed three different patterns, with north China having influenza peaks during winter months, whereas south China with peaks in July-September and cities around 30°N latitude with semi-annual peaks.[18] Further, recent published data from south and south-east countries also revealed a geographic influence on influenza seasonality, with countries in the tropical Asian belt (Bangladesh, Cambodia, Laos PDR, the Philippines, Thailand, and Viet Nam) having peak influenza activity during the monsoon months of July-September, whereas countries closer to the equator (Malaysia, Singapore, and Indonesia) had year round circulation.[9]Cumulatively, these data suggest that influenza circulation at the sub-regional level in subtropical regions of India and China at approximately ≥30° latitude have seasonality similar to northern temperate seasonality, whereas that below <30° latitude, influenza circulation is primarily during seasonality in rainy season. Thus, despite being in northern hemisphere, three distinct seasonality could be identified at the sub-regional level in India. The unpredictably of circulating strains across cities or years was clearly evident as both influenza A and B viruses and their subtypes co-circulated throughout the surveillance period across all cities. Overall, seasonal influenza A/H1 viruses were replaced by influenza A/H1N1pdm09 viruses in 2009, which persisted through 2010 and recurred in 2012 and persisted through 2013. These patterns of pandemic H1N1 circulation are comparable to what has been observed globally.[20] Influenza A/H3N2 virus was the predominant influenza A virus circulating in 2011 and 2013 in most cities. Our observation of rapid replacement of circulating influenza A/H1N1pdm09 virus in 2011 by influenza A/H3 virus has also been observed in other parts of the world.[21]Influenza B co-circulated through the study period in most cities to varying proportions; similar to what has been observed in other countries.[22] However, there was a surge in influenza B in 2012 coincident with circulation of the Yamagata lineage which led to selection of the Yamagata-like clade 2 virus in 2013–2014 northern hemisphere vaccine.[23]

The complex pattern of influenza circulation poses challenges for influenza vaccination timing. Thus Srinagar with winter temperate seasonality should consider vaccination in November-December, Chennai and Vellore at the south-eastern of India should consider vaccination in October-November, whereas rest of the country should consider vaccination in April-May (Table 2). Similar recommendations have recently been made for China.[18] Recent data from south and south-eastern countries in Asia also suggested that countries including Bangladesh, Cambodia, India, Laos PDR, Philippines, Thailand, and Vietnam should consider vaccination in May-June which precedes the seasonal epidemics in these countries using the most recent recommended vaccine.[9]

The current study has several limitations. First, the surveillance data collects a limited number of ILI cases every week in few clinics per site, which may not be representative of the whole city or state. However, consistent surveillance over several years provides a good overview of influenza peaks with circulating influenza types and subtypes. In fact, some of the states presented in the current study have population size equivalent to some countries, i.e. Uttar Pradesh in population size is equivalent to Brazil and Maharashtra is equivalent to Mexico. Additional surveillance sites from outpatient and inpatient hospitals are needed to better define the temporal and spatial distribution of circulating strains. While we are able to suggest a latitude gradient where temperate and tropical patterns for influenza peaks diverge, more robust data, especially from the northern part of India, are needed to define seasonality and latitude differences for influenza circulation patterns.

In summary, we show two main patterns of influenza circulation despite a wide range of environmental and latitudinal characteristics across India. We suggest that influenza vaccine policy in India should look closely at the circulation patterns in various states in India. These data suggest that cities above 30° latitude and those with late monsoon season (Chennai and Vellore) can continue winter vaccination strategies; however those below 30° should consider vaccination in May-June. In both cases the most recent WHO vaccine formulation should be used. These data should help policy makers to implement the strategy for choice of vaccine formulation and timing that is best suited for different regions of India.

### Ethics Committee approval

The protocol was approved by the Ethics Committees for research on human subjects at the participating sites viz. National Institute of Virology, Pune; Sheri-Kashmir Institute of Medical Sciences, Srinagar, Jammu & Kashmir; All India Institute of Medical Sciences, New Delhi; National Institute of Cholera and Enteric Diseases, Kolkata; Regional Medical Research Centre, Dibrugarh; King Institute of Preventive Medicine & Research, Chennai; Christian Medical College, Vellore; Indira Gandhi Medical College, Nagpur; King George Medical University (KGMU), Lucknow; National Institute of Virology, Alappuzha before commencement of the surveillance. Written informed consent was obtained from all study participants before enrolment. US-CDC considers surveillance and detection activities for influenza as non-research.

## Supporting Information

S1 Table Antigenic and Genetic similarities of A/H3N2 with vaccine selected strains. (DOCX)

S2 Table Antigenic and Genetic similarities of influenza B with vaccine selected strains. (DOCX)

S1 Fig Phylogenetic trees of virus isolates. A. Phylogenetic analysis of HA1 gene of seasonal A/H1N1 isolates. B. Phylogenetic Analysis of HA1 gene of A/H3N2 isolates. C. Phylogenetic Analysis of HA1 gene of A/H1N1pdm09 isolates. D. Phylogenetic Analysis of HA1 gene of Type B isolates. Taxon names are color coded- Black for 2009 isolates, Blue for 2009 isolates, Green for 2011 isolates, Dark Red for 2012 isolates, Pink for 2013 isolates.
(PDF)
