# Supplementary material for: Dynamics of Influenza Seasonality at Sub-Regional Levels in India and Implications for Vaccination Timing
Source: PLoS One. 2015 May 4;10(5):e0124122. doi: 10.1371/journal.pone.0124122 (PMC4418715; doi:10.1371/journal.pone.0124122)
Supplement: S1 Table — (DOCX) [file pone.0124122.s001.docx]

**Supplemental Information:**

**Table S1: Antigenic and Genetic similarities of A/H3N2 with vaccine selected strains**

| Year | H3N2 Vaccine  Components (SH)* | Antigenic Characterization  (HAI based)* | Genetic characterization (HA based) | Vaccine  Components (NH)* |
| --- | --- | --- | --- | --- |
| 2009 | A/Brisbane/10/2007 | Brisbane; n=110 | Perth; 102/112 (90.4%)  Brisbane; 10/112 (8.8%) | A/Brisbane/10/2007 |
| 2010 | A/Perth/16/2009 | A/Perth; n= 6 | A/Perth; 13/19 (70%)  A/Brisbane (Chennai) 6/19 (30%) | A/Perth/16/2009 |
| 2011 | A/Perth/16/2009 | A/Perth; n= 41 | A/Victoria; 36/37 (97.3%)  A/Brisbane (Chennai) 1/37(2.7%) | A/Perth/16/2009 |
| 2012 | A/Perth/16/2009 | A/Victoria; n=6 | A/Brisbane(Chennai); 7*/10(63.6%)  A/Victoria; 3/10 (27.3%) | A/Victoria/361/2011 |
| 2013 | A/Victoria/361/2011 | A/Victoria; n=58 | A/Texas; 39/48 (76.2%)  A/Brisbane(Chennai) 5/48(14.3%)  A/Victoria; 3/48(7.1%)  A/Perth (Delhi); 1/48(2.4%) | A/Victoria/361/2011 |
|  |  |  |  |  |

*****SH: Southern Hemisphere; NH: Northern Hemisphere; HAI: Haemagglutinin inhibition
