# Supplementary material for: Dynamics of Influenza Seasonality at Sub-Regional Levels in India and Implications for Vaccination Timing
Source: PLoS One. 2015 May 4;10(5):e0124122. doi: 10.1371/journal.pone.0124122 (PMC4418715; doi:10.1371/journal.pone.0124122)
Supplement: S2 Table — (DOCX) [file pone.0124122.s002.docx]

**Table S2: Antigenic and Genetic similarities of influenza B with vaccine selected strains**

| Year | Influenza B Vaccine  Components (SH) | Antigenic Characterization  (HAI based) | Genetic characterization (HA based) | Vaccine  Components (NH) |
| --- | --- | --- | --- | --- |
| 2009 | B/Florida/4/2006 | B/ Brisbane/60; n= 24  B/ Florida/4; n=2 | B/ Brisbane/60; 12/14 (85.7%)  B/Wisconsin; 2/14 (14.3%) | B/Brisbane/60/2008 |
| 2010 | B/Brisbane/60/2008 | B/ Brisbane/60; n=73  B/ Florida/4; n=16 | B/ Brisbane/60; 149/165(90.3%)  B/Wisconsin; 16/165(9.7%) | B/Brisbane/60/2008 |
| 2011 | B/Brisbane/60/2008 | B/ Brisbane/60; n= 61  B/ Florida/4; n=30 | B/ Brisbane/60; 34/34(100%) | B/Brisbane/60/2008 |
| 2012 | B/Brisbane/60/2008 | B/ Brisbane/60; n=60  B/Wisconsin; n=30 | B/ Brisbane/60; 29/34(85.3%)  B/Wisconsin; 1/34(2.9%)  B/Massachusetts; 4/34(11.8%) | B/Wisconsin/1/2010 |
| 2013 | B/Wisconsin/1/2010 | B/ Brisbane/60; n=10 B/Wisconsin; n=8 | B/ Brisbane/60; 2/4(50%)  B/Wisconsin; 1/4(25%)  B/Massachusetts; 1/4 (25%) | B/Massachusetts/2/2012 |
