# Supplementary material for: Dynamics of Influenza Seasonality at Sub-Regional Levels in India and Implications for Vaccination Timing
Source: PLoS One. 2015 May 4;10(5):e0124122. doi: 10.1371/journal.pone.0124122 (PMC4418715; doi:10.1371/journal.pone.0124122)
Supplement: S1 Fig — A. Phylogenetic analysis of HA1 gene of seasonal A/H1N1 isolates. B. Phylogenetic Analysis of HA1 gene of A/H3N2 isolates. C. Phylogenetic Analysis of HA1 gene of A/H1N1pdm09 isolates. D. Phylogenetic Analysis of HA1 gene of Type B isolates. Taxon names are color coded- Black for 2009 isolates, Blue for 2009 isolates, Green for 2011 isolates, Dark Red for 2012 isolates, Pink for 2013 isolates. (PDF) [file pone.0124122.s003.pdf]

Figure S1-A. Phylogenetic analysis of HA1 gene of seasonal A/H1N1 isolates.

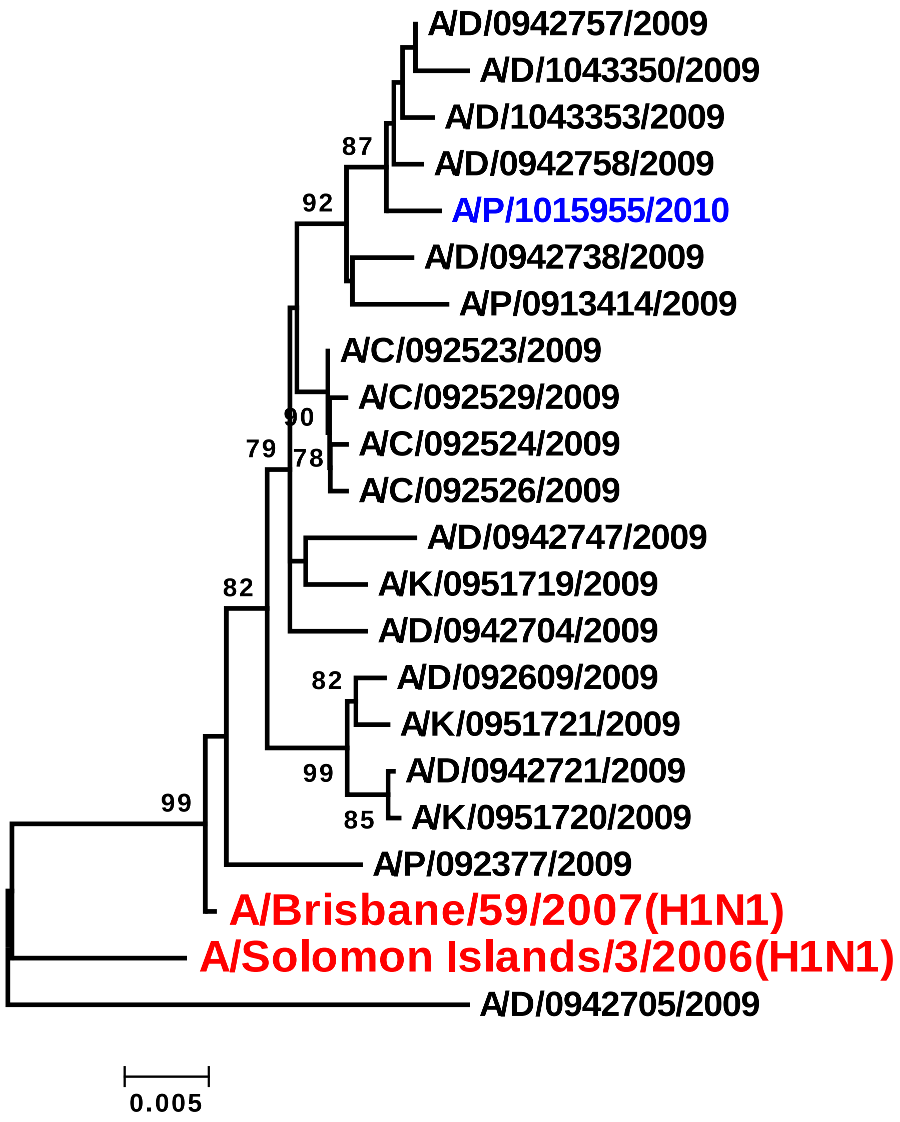

Figure S1-B. Phylogenetic Analysis of HA1 gene of H3N2 isolates

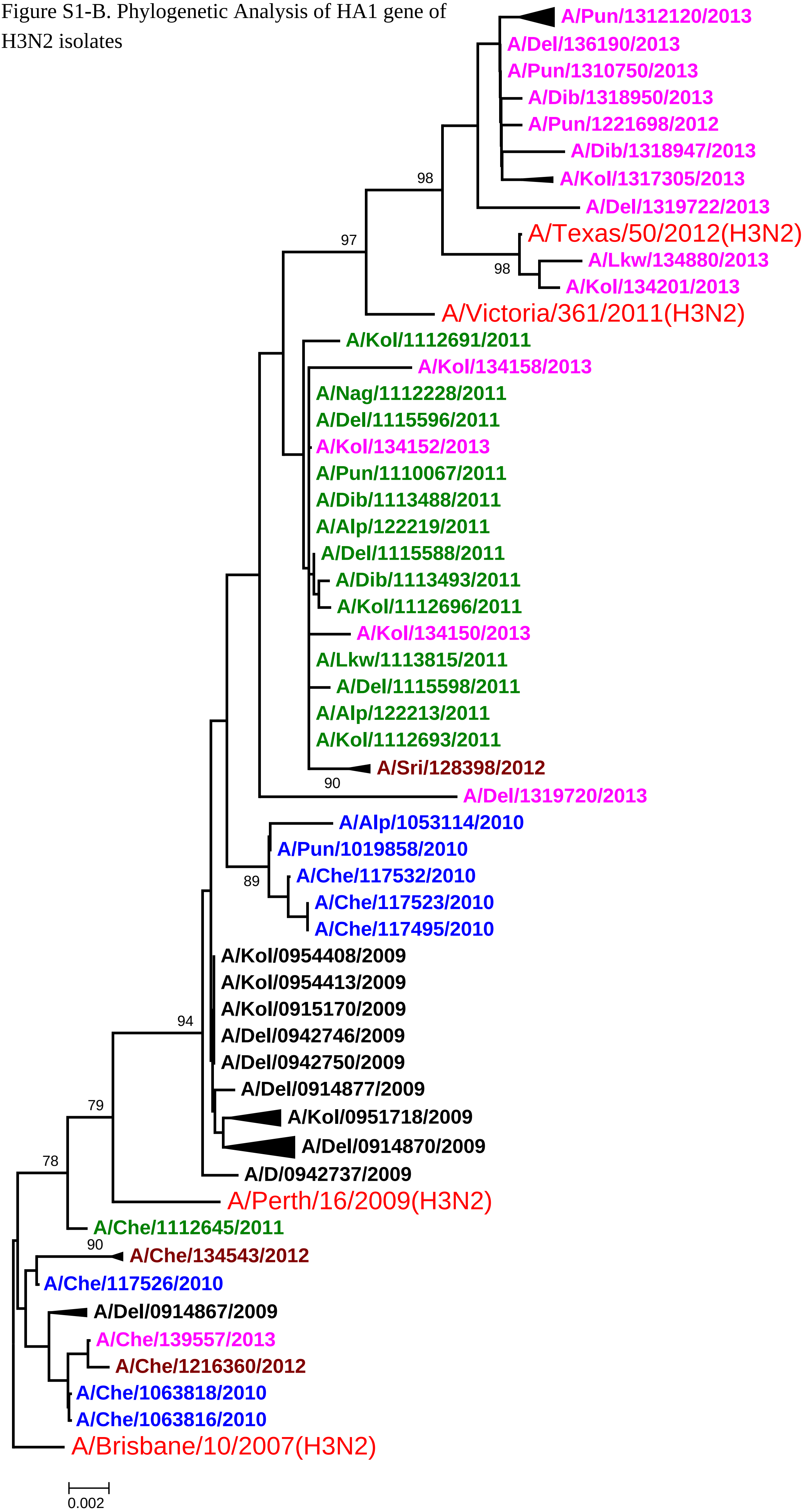

Figure S1-C. Phylogenetic Analysis of HA1 gene of A/H1N1psm09 isolates.

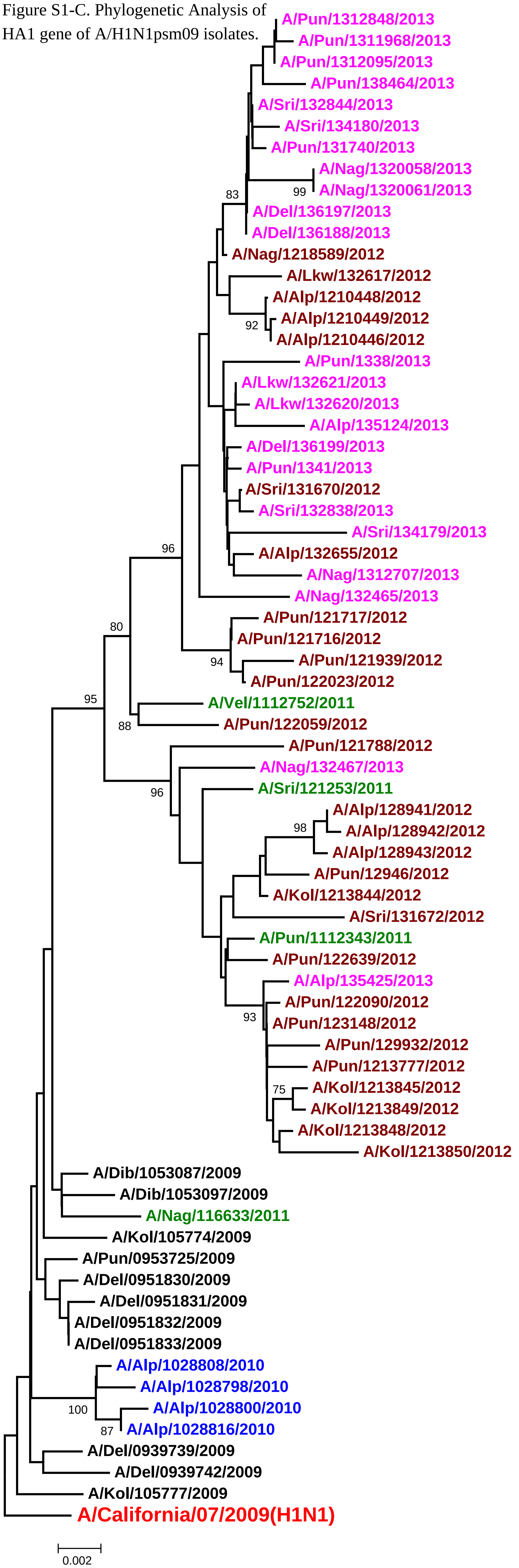

Figure S1-D. Phylogenetic Analysis of HA1 gene of Type B isolates.

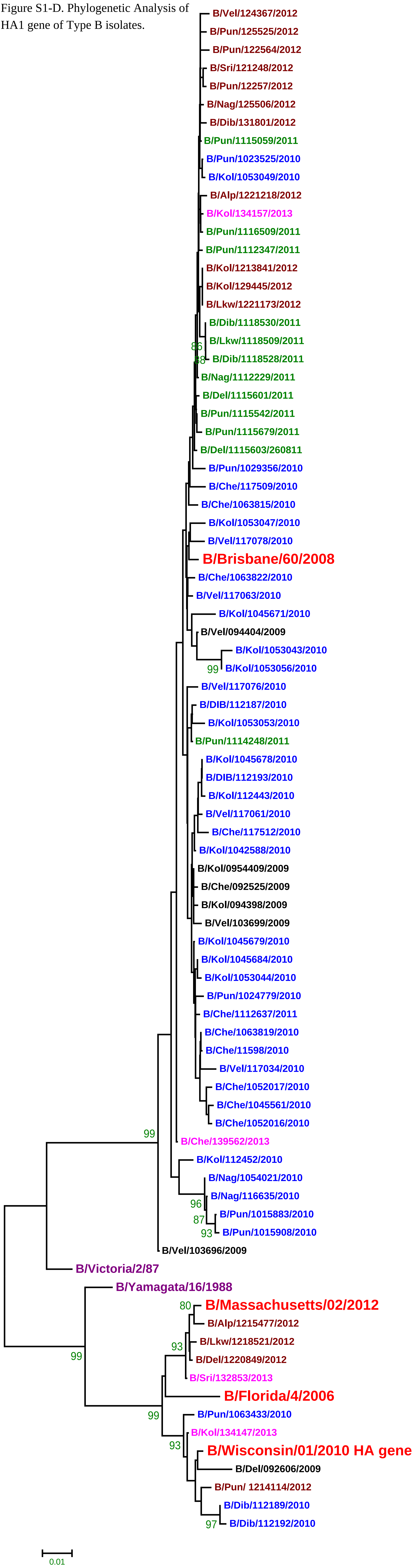

# Details of phylogenetic trees

A. Phylogenetic Analysis of HA1 gene of seasonal A/H1N1 isolates

B. Phylogenetic Analysis of HA1 gene of A/H3N2 isolates

C. Phylogenetic Analysis of HA gene of A/H1N1pdm09 isolates

D. Phylogenetic Analysis of HA1 gene of Type B isolates

## Taxon Name Color Coding

Vaccine components

2009 isolates, 2010 isolates, 2011 isolates, 2012 isolates, 2013 isolates
